# Evaluating the vulnerability of critical early life stages in plants during heat extremes

**DOI:** 10.1093/conphys/coag015

**Published:** 2026-03-27

**Authors:** Pieter A Arnold, Tara J Walker, Ella V Wishart, Lydia K Guja

**Affiliations:** Division of Ecology and Evolution, Research School of Biology, The Australian National University, 46 Sullivans Creek Road, Canberra, ACT 2600, Australia; Division of Ecology and Evolution, Research School of Biology, The Australian National University, 46 Sullivans Creek Road, Canberra, ACT 2600, Australia; Division of Ecology and Evolution, Research School of Biology, The Australian National University, 46 Sullivans Creek Road, Canberra, ACT 2600, Australia; Centre for Australian National Biodiversity Research (a joint venture between CSIRO and Parks Australia), CSIRO, GPO Box 1700, Canberra, ACT 2601, Australia; National Seed Bank, Australian National Botanic Gardens, Parks Australia, Canberra, ACT 2601, Australia; Umwelt Environmental and Social Consulting Canberra, Unit 3, 2-6 Shea Street, Phillip ACT 2606, Australia

**Keywords:** Drought, extreme climatic event, heat, heatwave, reproduction, thermal death time, thermal load sensitivity

## Abstract

Plants, their seeds and their gametes show remarkable resilience and responsiveness to environmental conditions. However, worsening climate change with more severe and frequent extreme climatic events, like heatwaves and hot droughts, will likely push many species towards or beyond their physiological limits. If such events occur during important points of development and reproduction—rather than mature vegetative growth—the direct impact on individual fitness can be high, with potential to bottleneck recruitment in populations. Here we take an ecophysiological perspective to discuss what is known of the effects of extreme heat on four critical early life stage transitions in wild plant development that affect fitness and recruitment. These life stages are pollen development, pollen germination to seed set, dormant-to-imbibed seeds and seed-to-seedling transition. We use the recently developed thermal load sensitivity framework to showcase how these critical points of ontogeny could be exposed to vastly different microclimate conditions and have different physiological heat tolerance. Assessing sensitivity of these life stage transitions to increasing thermal load with the additional stressors of limited soil moisture and drying atmosphere could be an effective approach to identify at-risk populations or species. We argue that vulnerable developmental stages and narrow reproductive windows that affect recruitment must be considered for effective conservation and restoration of plant populations under climate change.

## Abbreviations

ROS: Reactive oxygen species
TDT: Thermal death time
TLS: Thermal load sensitivity
VPD: Vapour pressure deficit

## Introduction

Extreme climatic events are now occurring more frequently across Earth with far-reaching impacts for life (Perkins-Kirkpatrick and Lewis, 2020; Thakur et al., 2022; Perkins-Kirkpatrick et al., 2024). Against a long-term background of steadily increasing average temperatures through climate warming, extreme heat events including individual hot days can act as ‘pulses’ that punctuate the impact of the rising ‘press’ of anthropogenic climate change (Harris et al., 2018). The severity of extreme heat events increases hyper-allometrically with average warming, meaning that expected extremes in the future are greater than a simple increase above background temperatures (Seneviratne et al., 2021).

The effects of extreme heat events like hot droughts and heatwaves are most often studied on adult populations of standing vegetation without considering flow on impacts to reproduction and recruitment of future populations. Yet, while extreme heat events are inherently rare occurrences (Perkins and Alexander, 2013), they are powerful forces for selection at life stages that have limited capacity to buffer against intense or repeated thermal stress (Buckley and Huey, 2016). Drought also often co-occurs with extreme heat due to precipitation deficits and changes in evapotranspiration that lead to lack of sufficient soil moisture. As temperature increases, the vapour pressure deficit (VPD) climbs exponentially, leading to greater atmospheric evaporative demands and amplification of drought stress (Grossiord et al., 2020; Novick et al., 2024). All these stressors represent significant challenges for physiological functions. Mortality of mature plants obviously limits potential recruitment into populations. Yet the impacts of drought, heat and/or high VPD on the production of viable seed and seedling establishment are more limiting in wild plants, with profound implications for population persistence.

### The impact of heat extremes and their timing on recruitment

There is high potential for recruitment failure at early life stages facing extreme heat. Reproductive and newly developing tissues are typically more susceptible to heat failure than vegetative tissues in plants (Ladinig et al., 2015, Lohani et al., 2020, Tushabe and Rosbakh, 2025). In many ecosystems, there are times of the year that are typically optimal for plant growth and reproduction, referred to here as the growing season (which may not be in consecutive months). This period often coincides with gradually increasing daylength, adequate soil moisture from rainfall or snowmelt and release from cold limitation by warmer conditions facilitating growth. Germinating seeds and seedlings can be pushed beyond physiological tipping points from extremes occurring early in the growing season (Niu et al., 2014; Fernández-Pascual et al., 2019; Cinto Mejía and Wetzel, 2023). Phenology—the timing of biological events—can be highly responsive to average warming, where earlier germination and earlier flowering are typical responses among plants (Anderson et al., 2012;  Parmesan and Hanley, 2015; Wadgymar et al., 2018). However, there is substantial risk that extremes that occur ‘unexpectedly’ (e.g. early in the growing season) will disproportionately impact reproductive fitness and more vulnerable early life stages (Cinto Mejía and Wetzel, 2023). Plants with predictable growing seasons may be more at risk from out-of-season (a-seasonal) extremes due to the phenology of physiological stress tolerance conferral matching seasonal change (Grossman, 2023).

Here we contend that research on the effects of heat at critical early life stages needs attention in wild plants. We briefly discuss developmental stages and physiological transitions that are vulnerable to heat and drought stress during a typical plant’s life cycle: (1) pollen development, (2) pollen germination to seed set, (3) dormant-to-imbibed seeds and (4) seed-to-seedling transition. We discuss how physiology can build understanding of the sensitivity of these important stages to extremes, focusing on heat (and not the potentially damaging effects of cold temperatures). We then use the thermal load sensitivity (TLS) modelling framework to showcase potential impacts of heat on these life stages. Effective conservation requires that we understand the effects of heat not only on adult vegetation, but also across other life stages. Conservation and restoration efforts can likely be enhanced by considering heat sensitivity from a whole-life-cycle perspective to develop predictive and protective approaches during establishment and reproduction periods.

## Key life stages for fitness are less resilient to heat stress

### Pollen development

Pollen develops through a complex, multi-stage process that requires coordinated function of multiple tissues (Hafidh et al., 2016). The complex nature of pollen development means it is easily disrupted by abiotic stress, ultimately causing fewer viable pollen grains to be released (Chaturvedi et al., 2021; De Storme and Geelen, 2014). Heat or drought stress, in either isolation or combination, during flowering can result in abnormal anther morphology and function (Rang et al., 2011; Tushabe et al., 2025); altered carbohydrate metabolism and inadequate nutrient reserve accumulation in pollen (Pressman et al., 2002; Hu et al., 2020); and oxidative damage to reproductive tissues (Djanaguiraman et al., 2018). When prolonged, even relatively mild increases in growth temperature can drastically reduce pollen viability and the number of grains released in tomato (Sato et al., 2006). Short periods of extreme stress can cause defects in mature pollen by disrupting specific processes, for example, chromosome segregation and cytokinesis during meiosis (De Storme and Geelen, 2020; Pécrix et al., 2011) and the timing of tapetal cell degeneration, which is crucial for normal pollen development (Saini et al., 1984). Stress exposure during early pollen development often results in greater reductions in pollen production and viability compared to later stages (Begcy et al., 2019; Chaturvedi et al., 2021).

**Figure 1:**
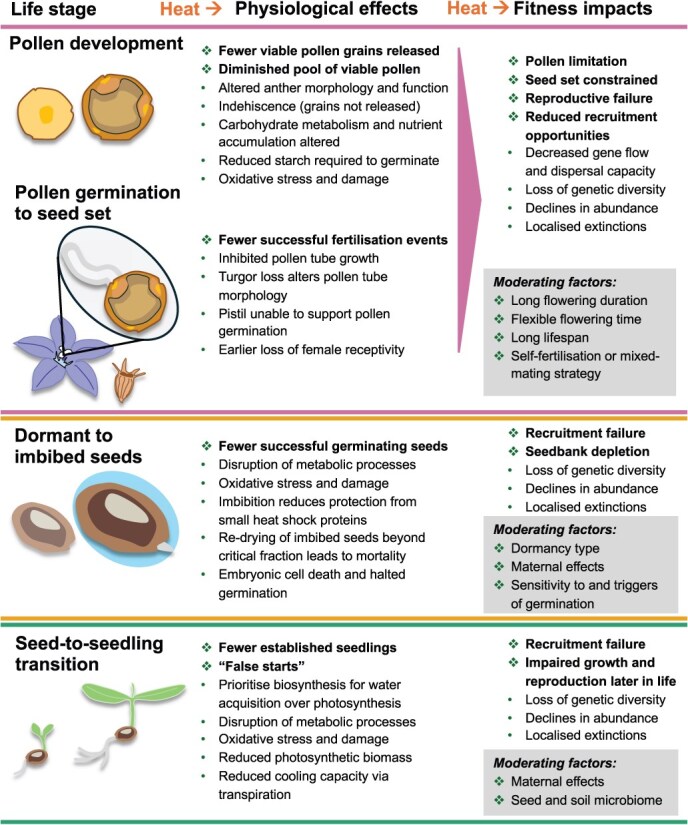
Four critical life stage transitions during a typical plant’s development. Some of the major (non-exhaustive) effects of extreme heat (with associated drying and high VPD) on physiological changes at each stage, and the fitness impacts for individuals and populations. Some moderating factors that could affect the impacts of heat on fitness are inset in grey boxes. Note that pollen development and pollen germination to seed set are grouped together to simplify fitness impacts and the coloured horizontal dividing lines match the colours of life stages used in Figs 2 and 3.

By limiting the number of viable pollen grains available, stress during pollen development drastically diminishes the chances of successful fertilization, with subsequent impacts on the success of sexual reproduction (Fig. 1). For fertile seed to be produced, viable pollen must be transferred to a compatible stigma and then germinate to deliver sperm cells to the ovule—all within a crucial window in which female tissue remains receptive (Hedhly et al., 2009). Very few of the pollen grains produced ever adhere to a compatible stigma in outcrossing species (Harder and Johnson, 2008), and subsequent pollen germination is both competitive and highly sensitive to abiotic conditions (Hedhly et al., 2009; Stokes and Geitmann, 2025). Reduced availability of viable pollen due to stress-affected development constrains the number of possible fertilization events. As such, heat stress contributes to and exacerbates pollen limitation—whereby seed production is limited by the quantity and quality of pollen grains reaching the stigma (Ashman et al., 2004; Rosenberger et al., 2024). Low seed set stemming from inviable pollen production contributes to the rarity of several threatened plant species (Kimpton et al., 2002; Burne et al., 2003), and it has implications for population persistence, gene flow and species distributions (Fernández-Pascual et al., 2019; Harder and Aizen, 2010).

### Pollen germination and seed set

Upon adhering to a compatible and receptive stigma, viable pollen rehydrates and germinates. It then forms a pollen tube that grows rapidly through the style and ruptures once reaching the embryo sac of an ovule to deposit sperm cells for fertilization (Firon et al., 2012; Johnson et al., 2019). Abiotic stress can reduce fertilization success by inhibiting pollen tube growth and limiting the ability of the pistil to support pollen germination (Hedhly et al., 2009; Snider et al., 2011). Pollen germination and the rate of pollen tube growth increase with temperature up to an optimum, then rapidly decline beyond a thermal maximum where tube growth fails (Kakani et al., 2002; Kakani et al., 2005; Steinacher and Wagner 2012). This thermal maximum is less than 45°C in many plant species and can be as low as 30°C (Stokes and Geitmann, 2025; Tushabe and Rosbakh, 2025). High temperatures are associated with increased reactive oxygen species (ROS) production in pollen tubes, leading to growth arrest or premature rupture (Muhlemann et al., 2018), altered respiration rates (Karapanos et al., 2009) and altered cell wall synthesis due to disturbed cytoskeletal function and anomalous enzyme distributions (Parrotta et al., 2016). Heat stress can also shorten the receptivity period of the stigma (Hedhly et al., 2005) and reduce the availability of soluble carbohydrates in the pistil to sustain pollen tube growth (Snider et al., 2011). Heat coupled with drought can cause osmotic stress from dehydration of female tissues that alters the volume and morphology of pollen tubes (Zonia and Munnik, 2004; Qian et al., 2025).

Declines in fertilization success caused by stress-impaired pollen germination can constrain the production of fertile seed (Snider and Oosterhuis, 2011). This limits opportunities for recruitment, with cascading ecological and evolutionary consequences for populations (Rosbakh et al., 2018) (Fig. 1). Short-lived species are also likely to be more affected by pollen-driven declines in seed set, since adults have fewer opportunities to reproduce over their lifetime (Iler et al., 2021). Self-compatible species may be less vulnerable to pollen limitation and subsequent reproductive failure than self-incompatible species (Busch and Schoen, 2008). Though self-incompatibility can be beneficial for adaptation and avoiding inbreeding, the inability to self-fertilize ultimately means self-incompatible plants have fewer sources of compatible pollen and a greater reliance on pollinator activity, both of which can be detrimentally impacted by abiotic stress (Burd, 1994; Busch and Schoen, 2008). Seed limitation can exert a strong influence on the growth and dynamics of wild plant populations (Ehrlén et al., 2006; Clark et al., 2007; Price et al., 2008). Lack of viable seed set due to impaired pollen tube growth and failed fertilization can then set population boundaries, lead to localized extinctions and alter community composition (Pigott and Huntley, 1981; Rosbakh et al., 2018). As pollen is a vessel for gene transfer, fertilization can shape the genetic structure and adaptation in subsequent generations (Ravikumar et al., 2003; Rounds et al., 2011). Impaired pollen availability and performance due to abiotic stress may also impact the capacity for plants to respond to future abiotic regimes (Hedhly et al., 2009).

### Dormant-to-imbibed seeds

In seeds with physical dormancy, a water-impermeable seed coat keeps the seeds in an anhydrobiotic state until dormancy break occurs (Baskin and Baskin 2014). Anhydrobiosis allows orthodox seeds to dry and survive in a dehydrated, dormant state where metabolic activity has declined to near zero, and long periods of drought and extreme heat can be endured (Crowe et al., 1992; Matilla, 2022). Seeds with morphological and/or physiological dormancy have water-permeable seed coats and thus can readily imbibe water while still in a dormant state (Baskin and Baskin 2014). Recalcitrant seeds do not undergo anhydrobiosis and thus retain a higher moisture content while in a quiescent state.

For simplicity, we do not discuss recalcitrant seeds in depth here, but it is worth noting two ways in which these broad categories differ in terms of vulnerability. First, the retained moisture content of recalcitrant seeds likely confers greater thermal vulnerability from dispersal until germination than orthodox seeds. Second, recalcitrant seeds are present in the soil seed bank for a limited time, where they germinate readily in high numbers and often persist as a seedling bank (Pammenter and Berjak, 2000). Therefore, the risk of exposure to extremes at a given life stage likely differs depending on ecological and evolutionary history. The lead up to imbibition also differs among seed types. Recalcitrant seeds maintain a high seed moisture content relative to orthodox seeds. Orthodox seeds undergo anhydrobiosis and can (i) persist in a state of quiescence prior to imbibition (non-dormant seeds); (ii) remain in a dry state until dormancy is alleviated, transferring them to a quiescent state prior to imbibition (all physically dormant seeds); or (iii) readily imbibe water while still in a dormant state.

Imbibition is the process that reverses anhydrobiosis, rehydrating the seed and facilitating repair of damage incurred during prolonged anhydrobiosis to allow germination to progress. The germination process is often described in three phases (Nonogaki et al., 2010; Bewley et al., 2013). Briefly, the phases are as follows: Phase I—imbibition is the first step in the germination process and involves cell rehydration and expansion; Phase II—an uptick in cellular respiration and the resumption of various metabolic processes (e.g. restoration of cellular integrity, and mitochondrial and DNA repair) that had been suspended or reduced prior to imbibition; Phase III—germination is complete once the radicle or other embryonic tissue has emerged from the seed coat.

Dry seeds can tolerate extreme temperatures before being detrimentally impacted (Bell and Williams, 1998; Hanley and Lamont, 2000; Ooi et al., 2014). However, if desiccation from drought or heat occurs during imbibition, this tolerance can be altered. Partial re-drying of imbibed seeds during the early stages of imbibition before reaching the critical fraction—beyond which the seed is committed to germination—can be favourable to harden seeds (Hanson, 1973). However, once beyond the critical fraction, orthodox seeds lose their desiccation tolerance and can no longer survive re-drying (Daws et al., 2007). Once seeds are imbibed, they become more vulnerable to abiotic factors including high temperature. Studies have typically explored high temperature effects on imbibed seeds in the context of fire-related soil heating (Ruprecht et al., 2016; Tangney et al., 2019). Nonetheless, we expect that extreme heat events would raise soil temperatures far above air temperatures (García-García et al., 2023), likely beyond physiological tolerance limits of imbibed seeds (Corbineau et al., 2002; Alvarez et al., 2023). This may be a result of available free water becoming heated to the extent that cellular machinery is damaged (Tangney et al., 2019). Additionally, small heat shock proteins may provide protection of key cellular machinery while seeds are in a dry state, but these reduce in abundance as seeds hydrate, thereby conferring less protection (Leprince et al., 2017). ROS play a crucial role in signalling throughout seed germination; however, heat stress can greatly increase ROS production and lead to substantial oxidative stress and cell death (Kranner et al., 2010; Gomes and Garcia, 2013).

While most dry seeds have a high thermal tolerance, the increase of extreme heat events (Perkins-Kirkpatrick and Lewis, 2020) and hazard-reduction burns (Agee and Skinner, 2005) occurring out-of-season when soil (and seed) moisture content is higher (Tangney et al., 2022) suggests that seeds will increasingly be exposed to high temperatures while in a vulnerable state. This could lead to widespread mortality that reduces the soil seed bank from which populations regenerate (Fig. 1). Seed and germination traits can adapt to the changing climate to shift towards trait values conducive to successful establishment, as long as the rate at which climatic changes occur does not outpace species’ adaptive capacity (Everingham et al., 2021). Water-impermeable seed coats may enhance persistence and thus shift community composition towards species that possess physical dormancy as a bet-hedging strategy (Pausas et al., 2022). Maternal resource provisioning and stress priming can be conferred to seeds to enhance seedling establishment (Brunel-Muguet et al., 2025). However, dormancy loss due to exposure to increased soil temperatures may diminish the extent to which physical dormancy can moderate the impact of extreme heat on seed bank persistence (Ooi et al., 2009; Cochrane, 2017). Finally, species that rely solely on regeneration from the soil seed bank may be more vulnerable to subsequent extreme events than species with re-sprouting ability. If a mortality event depleting the soil seed bank is followed by a disturbance event that diminishes the standing vegetation before adequate contributions are made to restock the seed bank, there is an enhanced threat of both immaturity risk and localized extinctions (Zedler et al., 1983; Keeley et al., 1999; Nolan et al., 2021).

### Seed-to-seedling transition

The seedling stage—referring to the period during which seed reserves are still partially utilized, ending when first true foliage is mature (Winkler et al., 2024)—is one of the most vulnerable stages of a plant’s development (Leck et al., 2008; Saatkamp et al., 2019). The seed-to-seedling transition marks a fundamental shift from heterotrophic to autotrophic metabolism (Henninger et al., 2022). Biosynthesis of chlorophyll to develop autotrophic competence is initiated by light and is highly responsive to environmental cues (Ha et al., 2017a). Both chlorophyll biosynthesis and response to abiotic stressors produce ROS, which can accumulate to cause oxidative damage and cell death (Mohanty et al., 2006; Ha et al., 2017a, 2017b), but are also essential for stress signalling (Mittler et al., 2011). The elongation of seedling shoot apical meristems and cotyledons occurs with exposure to warm temperatures (Lee et al., 2014), which can facilitate swift emergence through the soil and enable leaf thermoregulation by increasing distance from soil that radiates heat (Crawford et al., 2012). Developing seedlings need to rapidly expand to establish autotrophic growth, where biosynthesis within the cotyledons establishes photosynthetic machinery and begins carbon fixation while seed reserves are still being mobilized (Kandar and Pal, 2024).

Plants have evolved remarkable developmental plasticity in response to their environment (Sultan, 1995, 2000)—germinating seeds can slow metabolic processes to conserve energy, activate stress responses and delay the shift to autotrophic metabolism until conditions improve (Rosental et al., 2014). Nonetheless, ‘false starts’ occur due to sensitive germination response during temporarily favourable conditions that are followed by periods of stress. Abiotic stress, especially heat and drought, can quickly prove lethal to emerging seedlings or impair seedling growth with lasting fitness consequences (Dekkers et al., 2015; Smolikova et al., 2021; Winkler et al., 2024). As less predictable extremes occur more frequently and outside typical season patterns due to climate change progression, poorly timed germination could cause failure of entire cohorts of seedlings (Orsenigo et al., 2014; Fernández-Pascual et al., 2019). Early vs late extreme heat can have differential impacts on fecundity and survival of plants (Dreesen et al., 2015; Cope et al., 2023). Even arid dryland grasses that are highly adapted to taking advantage of narrow germination windows are threatened by both heat and water stress, such that extreme heat during water limitation can cause > 95% recruitment failure (Lewandrowski et al., 2021). Dry soil exposed to high solar input greatly exceeds air temperatures (Qiu et al., 1998), resulting in inescapable extreme heat conditions for small plants, which accumulate to exceed physiological tolerance thresholds (Kolb and Robberecht, 1996; Hankin et al., 2025). Prolonged dry conditions with concurrently high temperatures presents a lethal combination for seedlings well after initial establishment (Moran et al., 2019; Hankin et al., 2025).

Stressful conditions during development can diminish individual- and population-level fitness (Fig. 1). To endure extreme heat and drought, seedlings must slow their metabolism and prioritize biosynthesis for water acquisition over photosynthetic tissues. Consequently, these stressors early in plant development can lead to impaired growth and poor reproductive outcomes that reduce individual fitness (Zhang et al., 2008; Shevtsova et al., 2009; Hou et al., 2014; Winkler et al., 2019). Recruitment of seedlings is already a substantial bottleneck for populations due to their limited resources, vulnerability to biotic and abiotic factors and exposure to high competition (Eriksson and Ehrlén, 2008). Further, the microbiome of the seed and the soil it germinates in plays a potentially significant role in facilitating seedling establishment (Nelson, 2018). Ultimately, if cohorts of seedlings are unable to establish, or have reduced fitness later in life, then population persistence is likely to be jeopardized.

Seedlings that do manage to establish then enter an extended phase of vegetative growth, where they photosynthesize to accumulate biomass to support later reproductive phases. The vegetative stage is generally much more robust than the life stages discussed here due to established root systems and autotrophy. Further, leaves of vegetative plants are often numerous, and are replaceable modules rather than whole individuals (i.e. seeds and seedlings). While the thermal tolerance of mature vegetative tissue is outside the focus of this perspective, there is a vast literature for readers to explore further (Lancaster and Humphreys, 2020; Geange et al., 2021; Perez et al., 2025).

## Modelling TLS across vulnerable life stages

There are now sophisticated biophysical models to estimate plant temperatures through time based on high-resolution microclimate data sources (Kearney and Porter, 2020, Klinges et al., 2022; Meyer et al., 2023; Kearney and Leigh, 2024). Here we provide a simplified demonstration of potential ways to model probability of mortality or ‘failure’ due to heat at early life stages using biophysical models and physiological parameters within the TLS framework (Arnold et al., 2025a). Here, we use a location near Richmond, New South Wales, Australia (150.7379°E, 33.6187°S) for these illustrative examples. This location is classified as a humid subtropical climate but can have extreme heat, with record maximum temperatures ≥40°C between October and March, with a highest recorded 47.4°C in January 2020.

To model relevant environment conditions for the different life stages, we used the *micro_silo* implementation of the microclimate model in *NicheMapR* for Australia (Kearney and Porter, 2020). We simulated a generic small plant’s microclimate: for pollen at 50 cm, for imbibed seeds at the soil surface (0 cm) and for seedlings at 1 cm aboveground (relevant for newly emerged seedling height). For all simulations, we set a minimum of 0% shade and maximum of 60% shade to simulate a relatively thin canopy that could be expected in many ecosystems during periods of drought and heat. Solar radiation, rainfall effects on the soil moisture, relative humidity and wind speed, among others, are all modelled as part of the *micro_silo* implementation (Kearney and Porter, 2017). We fitted ‘ectotherm’ models in ‘leaf mode’, following the procedure outlined in Kearney and Leigh (2024). The same principles can be applied across life stages if different aspects of the plant’s microclimate are captured in the fitted parameters. For simplification purposes, we fitted the model for pollen as a single stage, along with imbibed seed (‘seed’) and seedling stages. All code to demonstrate the modelling procedures and to replicate the simulations is openly available (https://pieterarnold.github.io/Life_stage_TLS/).

**Figure 2:**
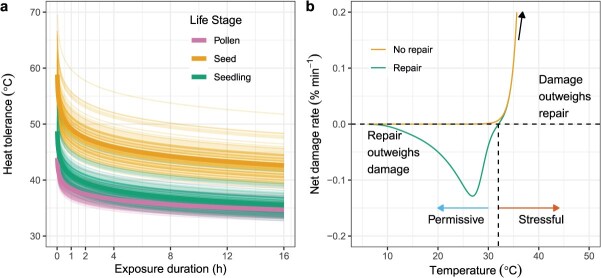
TLS modelling inputs. (**a**) TDT curves showing the relationship between heat tolerance and exposure duration across the three life stages that differ in heat tolerance and sensitivity. As exposure duration increases, heat tolerance declines but to different extents across the life stages. Thick lines show the mean population-level (*n* = 100) responses of the thin lines that are simulated individuals. (**b**) Example relationship between net damage and temperature, showing that for a given minute, repair outweighs damage below ~ 31°C (the ‘permissive’ range), while damage outweighs repair (the ‘stressful’ range) above it and damage accumulates exponentially, well beyond the maximum repair rate (black arrow indicates exponential increase).

Here we simulate individual- and population-level heat failure risk using reasonable initial estimates from our own preliminary data. Although ecophysiological data for seedlings is very limited, the heat tolerance limits of photosystem II in cotyledons and first leaves appear similar to that of adult plants (Alvarez et al., 2025). The key difference is that these tissues are essentially the entirety of productive biomass for seedlings, while they are a replaceable module of vegetative, mature plants. We fitted TLS (akin to thermal death time or TDT) models to the three life stages based to show how the highest temperature that each can tolerate (heat tolerance) differ but clearly decrease when exposed to that temperature for longer durations (Fig. 2a). For simulations of mortality through time, we calculate net damage based on the difference between a damage accumulation model (Ørsted et al., 2024) and an Arrhenius-type repair function (Arnold et al., 2025a). The net damage rate can then be separated into two parts. The first is a range of ‘permissive’ temperatures in which repair outweighs damage, resulting in net repair. Then, beyond the permissive range, temperatures are ‘stressful’ in which damage outweighs repair and damage accumulates exponentially (Ørsted et al., 2022) (Fig. 2b). Heat failure rate in the stressful range escalates extremely rapidly (>100% per 1°C) due to exceeding physiological tipping points (Jørgensen et al., 2022). Including repair allows heat failure probability to decline as damage is repaired during periods when temperatures are permissive.

**Figure 3:**
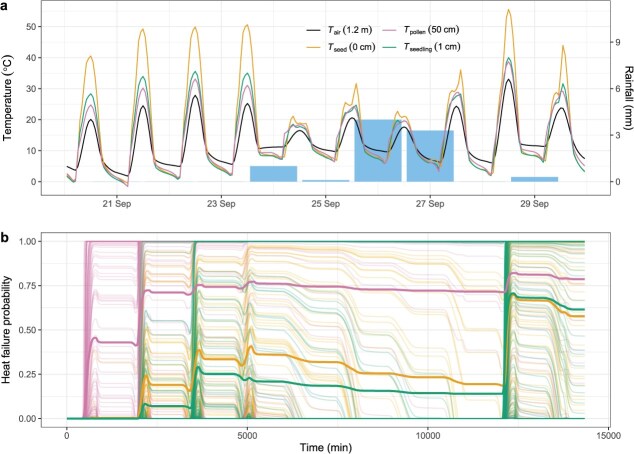
Simulated microclimate temperatures and heat failure probability across life stages. (**a**) Temperatures at different heights above the ground differ drastically, especially on hot days with high solar radiation input and without rainfall. *T*_air_ at 1.2 m is equivalent to standard air temperature weather forecasts. Taking the first day as an example, an imbibed seed right at the soil surface could reach temperatures 20°C above *T*_air_, while a seedling 1 cm above it could be much cooler but still 8°C above *T*_air_. (**b**) By modelling the damage accumulation over time and allowing for repair at permissive temperatures, we can estimate the heat failure probability at the individual (thin lines) and mean population levels (thick lines) among life stages across the time series. Due to the differences in TLS, each individual and life stage has a different heat failure probability under identical (within-stage) environmental conditions.

By simulating the microclimates at relevant vertical stratifications for the different life stages, we illustrate how radically different microclimate temperatures emerge from biophysical principles, especially on hot days (Fig. 3a). All life stages reach temperatures well above the standard 1.2-m air temperature, especially seeds and seedlings that are closest to the soil surface under high solar load (Fig. 3a). This approach allows us to model heat failure probability across the time series of microclimate temperatures by determining the hazard of heat accumulation over time (Fig. 3b). The notable spikes in heat failure probability align with the hottest daytime temperatures reached but clearly vary among the population (Fig. 3b) even with relatively minor differences in their sensitivity to heat exposure (i.e. Fig. 2a). Despite the much higher temperatures in the seed microclimate, their higher tolerance reduces heat failure probability to well below pollen and akin to seedlings at the end of the simulation (Fig. 3b). Here, the individuals that have heat failure probability <1 can repair damage during the cooler period with rainfall. However, those individuals that had relatively more damage remain more likely to succumb to accumulated damage, such that the recurrent heat event requires less damage to reach a physiological tipping point, for damage to accumulate and for heat failure probability to reach 1 (Fig. 3b).

We note that our illustrative example models are a starting point for generating hypotheses and stimulating ideas for further modelling and empirical science. They are generalizations of the biophysical forces acting on simplified structures with arbitrarily set properties. The discrete heights assume that seeds are germinating directly at the soil surface and simplified effects of sparse surrounding vegetation to simulate shade. Nonetheless, microclimate models are a vast improvement over assuming nearby air temperature is equivalent to plant temperature. The extreme thermal deviations and vertical stratification at biologically relevant fine scales cannot be overlooked anymore (Körner and Hiltbrunner, 2018; Klinges et al., 2025). Leaf heat tolerance is generally weakly predicted by species-level traits, phylogenetics and origin macroclimates (Perez and Feeley, 2021; Bison and Michaletz, 2024; Briceño et al., 2025). However, we now know that the thermal conditions to which plant tissues are directly exposed 4 days prior to tolerance assays can predict heat tolerance thresholds with relatively high accuracy (Pottinger et al., 2025). As these life stages occur sequentially, at the individual or population level, the cumulative hazard of heat failure may be additive across these developmental stages, exacerbating the impact of extremes on fitness.

Empirical validation of how damage and repair processes manifest to affect heat failure probability in early and reproductive life stages of plants needs to be investigated from biochemical and energetic perspectives (Sokolova, 2021; Buerger et al., 2023). The different scales and modularity of plants also need to be considered further. For example, a plant may produce hundreds to thousands of flowers with millions of individual pollen grains, so damage could be a proportion of grains rendered inviable or the number of flowers aborted per individual, while a seed or seedling is usually a discrete unit. Nonetheless, the procedural damage-repair approaches certainly offer an improvement over single point critical thresholds and thermal safety margin approaches that do not include exposure duration (Lutterschmidt and Hutchison, 1997; Ørsted et al., 2022; Cook et al., 2024; Arnold et al., 2025a). Integrating life stage-specific sensitivity among species using a systems modelling approach seems promising for developing understanding of potential community responses in complex ecological systems (Noble et al., 2026).

## Emerging themes for empirical inquiry

Clearly there needs to be empirical effort to explore damage and repair, and to ground-truth both thermal exposure through microclimates and TLS for key life stages in populations of interest, not just mature vegetation. There are many standardized protocols and guidelines for data collection of regenerative plant functional traits (Miller et al., 2017; Larson et al., 2025; Poschlod et al., 2025). We also need to evaluate the abiotic and biotic modifiers for TLS across life stages. For example, warming or drought (soil or atmospheric) conditions prior to an extreme heat event could improve heat tolerance from the upregulation of protective mechanisms via cross-tolerance, stress memory or priming (Hossain et al., 2018; Bryant et al., 2024; Harris et al., 2024; Rodgers et al., 2025).

Plants can display astonishing phenotypic plasticity in response to growth conditions (Nicotra et al., 2010; Arnold et al., 2019, 2024). Some species have capacity to rapidly upregulate heat tolerance in photosynthetic tissues in response to heat in hours to days (Havaux, 1993; Zhu et al., 2018, 2024; Andrew et al., 2023, 2024), even seedlings (Alvarez et al., 2025). However, inducible plasticity and eco-evolutionary responses to abiotic stressors will have costs and limits that are not well characterized for mature wild plants, let alone for early and reproductive life stages thereof (DeWitt et al., 1998; Hendry, 2016). Alternative strategies that limit heat transfer through passive structural traits and active thermoregulation rather than altering physiological heat tolerance could be effective for persisting through heat events, and the diversity of responses needs further exploration (van Zanten et al., 2021; Guo et al., 2025; Arnold et al., 2025b). Generating empirical data to validate parameters and simulations such as our example could facilitate forecasting heat failure probability, which can be integrated across life stages and systems based on both realized exposure and physiological limits (Noble et al., 2026).

## Conservation and restoration need physiologically resilient early and reproductive life stages

It is increasingly evident that meeting conservation goals will require consideration of how new plants can establish and build resilient, self-sustaining populations (Miller et al., 2017). The early and reproductive life stages discussed here are essential for the success of both retaining ecological values in currently conserved sites, and in re-establishing those values in degraded areas requiring active management and restoration (Tomlinson et al., 2022). Identifying the conditions that limit the establishment, growth and survival of plants at early life stages is fundamental. Understanding the ecophysiology of these systems will assist, even if the resultant actions of managers and practitioners differ depending on conservation or restoration context. Heat exposure is modified by the conditions of the plant’s surrounding micro-environment, physical, chemical, biotic and hydrological aspects of the soil substrate and the surrounding biota (Sharma and Kumar, 2023). We need to consider how natural or artificial structures (to add shade or protection) or treatments (addition of water, soil microbiota or pre-stress conditioning) can be applied to ameliorate heat exposure during key life stages at appropriate scales (Miller et al., 2017; Tomlinson et al., 2022).

The range of possible environments that support seed germination through to complete seedling establishment (i.e. regeneration niche breadth) needs greater attention. Experimental approaches like determining the thermal or hydrothermal germination niche of seeds for conservation or restoration can evaluate the window of opportunity for seed germination (Rajapakshe et al., 2024; Hirst et al., 2025). By integrating physiological windows such as these with microclimate windows from mechanistic niche mapping tools, we can generate heat failure probabilities for different life stages across time and space. Building spatiotemporal maps of heat failure probabilities based on the TLS framework has potential to integrate with spatial prioritization tools (Unnithan Kumar et al., 2025) to explore the effectiveness or feasibility of intervention treatments.

Restoration and conservation efforts require careful consideration of species and provenances of seeds and seedlings. For sustainable restoration and longer-term conservation, these need to be tolerant to the most extreme microclimatic conditions to which they are (and will be) exposed at key points of ontogeny, not just the macroclimatic averages (Gross et al., 2017; Miller et al., 2017). This is especially important in restoration of degraded landscapes that are inherently more open and less protected from extremes (Valliere et al., 2022). Ensuring that the chosen plants have reasonable likelihood to reproduce successfully also needs to consider the timing window of reproductive events and their sensitivity to extremes. Achieving sustainable conservation and restoration targets requires ecophysiology of early and reproductive life stages and extremes to be explicitly considered to increase long-term resilience at the plant, population and ecosystem level (Tomlinson et al., 2022; Valliere et al., 2022).

## Conclusion

Plants face a more extreme future that threatens individual- and population-level fitness. Here, we have highlighted the need for focus on early and reproductive life stages as key transitions throughout development. We have showcased how the evaluation of thermal exposure and TLS can lead to strong differential impacts of extreme heat across life stages. Physiology is central to understanding how biological entities and ecological systems will cope with the prolonged, intense heat and other extreme environmental perturbations in the future. Conservation and restoration efforts need to ensure that the vulnerability of early and reproductive life stages to climate extremes, more than averages, is considered and supported so that populations have high likelihood to establish and reproduce in the short term to be self-sustaining in the long term.

## Data Availability

No specific data are associated with this article; however, R code for simulations is openly available on GitHub at https://pieterarnold.github.io/Life_stage_TLS/ and Zenodo at https://doi.org/10.5281/zenodo.18707545.
